# Neoadjuvant PRRT for advanced pNEN: an unusual highlander

**DOI:** 10.1007/s12020-021-02662-9

**Published:** 2021-03-04

**Authors:** Isabella Zanata, Maria Rosaria Ambrosio, Maria Chiara Zatelli

**Affiliations:** Section of Endocrinology and Internal Medicine, Department of Medical Sciences, University of Ferrara, Ferrara, Italy

**Fig. 1:**
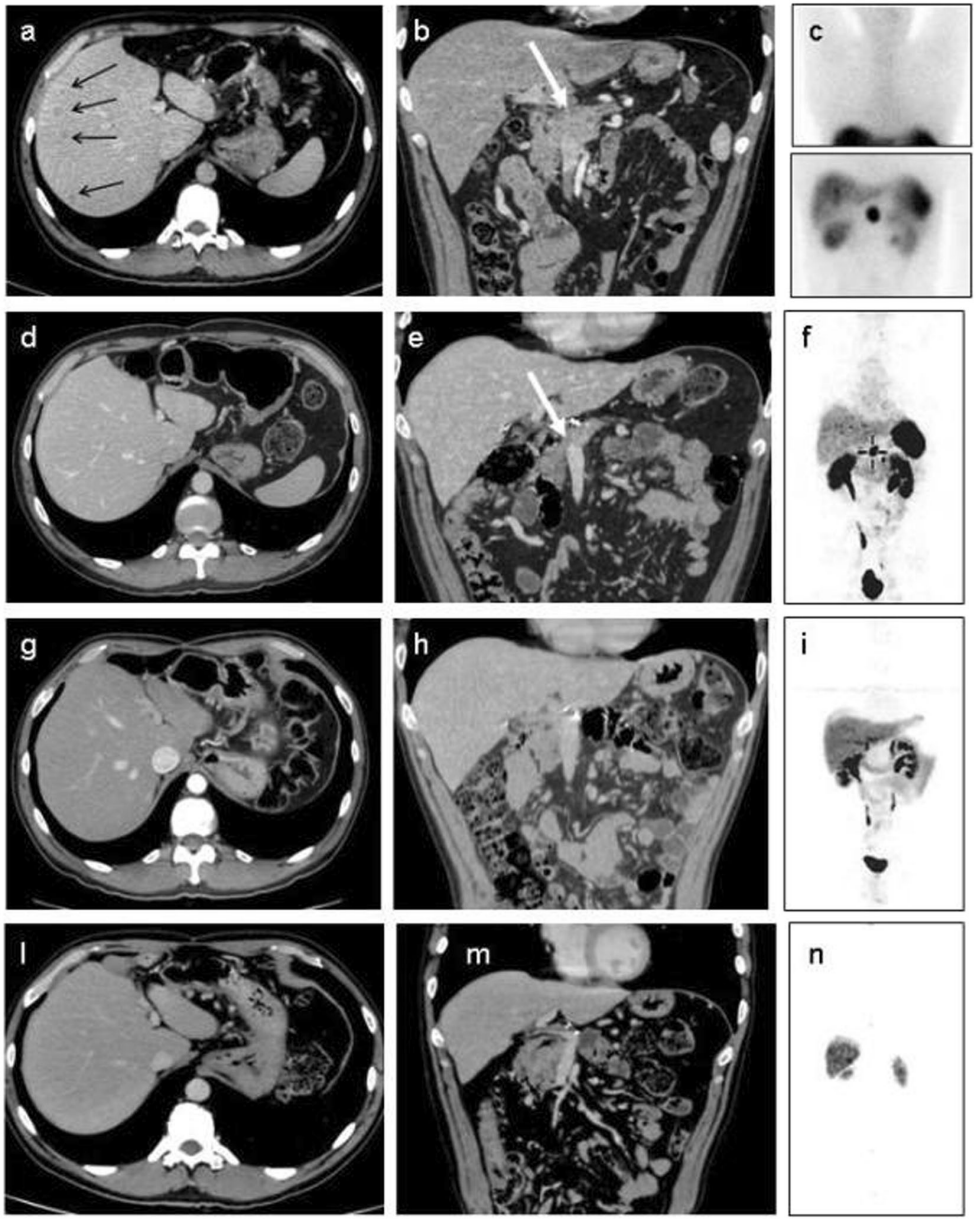
Clinical case imaging. Imaging at the time of diagnosis. Axial portal venous phase CT (**a**) shows multiple metastases in the right liver lobe (*arrows*). Multiplanar coronal reconstruction (**b**) shows a 4 cm mass (*white arrow*) in the pancreatic body. Anterior planar Octreoscan scan (**c**) confirms the pathological intense tracer uptake in the pancreatic tumor and liver metastases. The patient provided written informed consent for disclosing clinical and imaging data. Follow-up imaging 5 months after PRRT. Axial portal venous phase CT scan (**d**) shows the absence of liver metastases and the reduction (>50%) in the pancreatic mass (*white arrow*) (**e**). Anterior planar ^68^Gallium-DOTANOC PET-CT scan (**f**) confirms the absence of the pathological uptake in the liver and a radiotracer uptake reduction in the pancreatic tumor. **g–i** Imaging 3 months after surgery. Axial CT scan (**g**) shows post-operative findings of distal pancreatectomy and splenectomy with a persistent absence of liver metastases. On coronal CT image (**h**), the pancreas head is free of local recurrence. Anterior planar ^68^Gallium-DOTANOC PET-CT scan (**i**) confirms the absence of the pathological uptake both in the liver and in the residual pancreas. Imaging ~10 years after surgery. Axial-coronal CT scans (**l**, **m**) and anterior planar ^68^Gallium-DOTANOC PET-CT scan (**n**) overlap with the previous morpho-functional imaging and show the absence of recurrence both in the liver and in the residual pancreas

Neoadjuvant treatment may be useful in inoperable pancreatic neuroendocrine neoplasms (pNEN). A 41-year-old man complained for severe gastrointestinal symptoms (severe dysphagia, gastroesophageal reflux, postprandial diarrhoea, and weight loss); abdominal ultrasound showed two liver nodules and computerized tomography (CT) showed multiple liver metastases and a 4 cm highly vasularized pancreatic mass, suspected for NEN (Fig. 1a, b). Chromogranin A (CgA), Neuron Specific Enolase, gastrin, glucose and insulin levels, blood count, and liver, kidney, adrenal, and pituitary function were normal. Calcitonin levels were high (151 pg/ml). Somatostatin receptor (SSTR) scintigraphy (Octreoscan) showed a strong uptake in the pancreatic tumor and liver metastases (Fig. 1c); the patient started medical therapy with a long acting somatostatin analogue (SSA) in keeping with current guidelines [1] and with a strong Octreoscan positivity. Surgical exploration with multiple biopsies showed a locally invasive, well-differentiated G1 (Ki-67 index <2%) NEN with positive immunohistochemistry for calcitonin, CgA and SSTR subtypes 1, 2, 3, and 5 (pNEN cT_4_N_X_M_1A_; Stage IV), according to AJCC TNM classification (8th edition). Surgical management was contraindicated at that time due to portal and splenic vein involvement. A systemic neoadjuvant treatment was needed in order to manage the disease with the perspective of a future surgical treatment to allow tumor removal and prolong survival of this young patient. Everolimus and sunitinib are approved treatments after SSA failure, but were unavailable at that time. Cytotoxic chemotherapy is indicated as first-line therapy in G2 pNEN, which was not our case. Ablative loco-regional therapies could have addressed liver disease, leaving untreated the pancreatic mass [2]. Therefore, we opted for peptide receptor radionuclide therapy (PRRT), despite this approach is not routinely employed in neoadjuvant and adjuvant settings in pNEN, in keeping with the inclusion criteria indicated by the ENETS 2009 Consensus Guidelines [3]. PRRT is recommended in nonfunctional G1–G2 unresectable pNEN after failure of medical therapy and it may improve symptoms and quality of life in patients with functioning pNEN [4]. Our patient underwent five PRRT cycles with ^90^Yttrium (^90^Y)-DOTATOC (10.619 MBq cumulative dose in 9 months), without side effects. The patient was treated with Y-DOTATOC because that was the only PRRT treatment available at the time the patient was addressed to this approach.

Post PRRT abdominal CT showed a >50% reduction in pancreatic tumor size (maximum diameter from 4 to 1.3 cm) and disappearance of liver metastases (Fig. 1d, e), but persistence of splenic vein occlusion, as confirmed by ^68^Gallium-DOTANOC PET-CT (Fig. 1f). Calcitonin levels (2.7 pg/ml), blood glucose and insulin levels were normal. The case was discussed in the NEN tumor board and the patient was found eligible for distal pancreatectomy and splenectomy. Histopathology showed a 2 cm well-differentiated NEN with Ki-67 index = 1% (Grade 1) with perineural invasion (pNEN pT_1_N_0_M_0_, Stage I). Post-surgical abdominal CT showed the absence of liver metastases, portal vein invasion and pancreatic recurrence (Fig. 1g, h), as confirmed by ^68^Gallium-DOTANOC PET-CT (Fig. 1i). Calcitonin levels were undetectable (<1 pg/ml). Long-term yearly follow-up (>9 years) evaluated biochemistry, abdominal CT and ^68^Gallium-DOTATOC PET-CT and allowed to observe a progression-free survival (PFS) after neoadjuvant PRRT of > 108 months, which is threefold to fourfold longer as compared to previously observed results. PRRT indeed caused a tumor down-staging from Stage IV to Stage I, allowing complete surgical resection. Eight years after PRRT and 7 years after surgery, SSA therapy was stopped. Two years after SSA treatment withdrawal, no biochemical nor morpho-functional evidence of recurrence has been detected and the patient is free of disease 11 years after initial diagnosis (Fig. 1l–n).

Neoadjuvant PRRT has already been reported to reduce NEN bulk in cases that were not eligible for surgery at diagnosis [5–10] also in pNEN. Recently, PRRT efficacy was demonstrated in pNEN in neoadjuvant settings, showing that patients previously treated with PRRT have a lower incidence of nodal metastases at the time of surgery, reduced intra- and post-surgical complications and longer PFS (52 months) as compared to patients treated only with surgery (37 months) [11]. We found 6 studies describing a total of 21 patients undergoing neoadjuvant PRRT for an unoperable pNEN, who eventually underwent surgery obtaining a complete tumor resection (R0 resection) [5, 12–16].

Our clinical experience is in line with current literature on pNEN and proves the potential efficacy of neoadjuvant 90Y-PRRT in G1 metastatic pNEN. PRRT, indeed, may improve life expectancy and long-term prognosis after surgery, without major side effects. Current guidelines do not provide specific indication concerning adjuvant SSA therapy duration after PRRT: our case underlines the importance of a multidisciplinary evaluation in order to tailor the treatment according to patient’s characteristics.
